# Surveillance for Antimicrobial Resistance in Croatia

**DOI:** 10.3201/eid0801.010143

**Published:** 2002-01

**Authors:** Arjana Tambiæ Andraševiæ, Tera Tambiæ, Smilja Kaleniæ, Vera Jankoviæ, the Working Group of the Croatian Committee for Antibiotic Resistance Surveillance

**Affiliations:** * University Hospital for Infectious Diseases “Dr F. Mihaljeviæ,” Zagreb, Croatia; † Croatian Academy of Medical Sciences, Zagreb, Croatia; ‡ Zagreb Clinical Hospital Center, Zagreb, Croatia; § Croatian National Institute of Public Health, Zagreb, Croatia

**Keywords:** antimicrobial resistance, empirical therapy, surveillance

We describe the activities of the Croatian Committee for Antibiotic Resistance Surveillance and report surveillance results for 1999. Twenty-two Croatian microbiology laboratories participated in the study. Resistance rates for the organisms isolated in different centers varied widely, but certain trends were apparent. Penicillin resistance in pneumococci (38%), methicillin resistance in *Staphylococcus aureus* (22%), the production of extended spectrum beta-lactamases by *Klebsiella pneumoniae* (21%), and imipenem resistance in *Pseudomonas aeruginosa* (11%) represent major resistance problems, especially in large hospitals. A comprehensive system of antimicrobial resistance surveillance, combined with training and external quality control programs, has identified high rates of resistance in key pathogens in some regions of Croatia. The program has heightened awareness of the problems of antimicrobial resistance and contributed to ongoing improvements in laboratory practice.

Antimicrobial resistance is a well-recognized problem worldwide (*1*). Resistant organisms are more likely in intensive care settings (*2*), where a combination of debilitated patients, invasive technology, and high antimicrobial use facilitates infections by multidrug-resistant staphylococci, enterobacteria resistant to third-generation cephalosporins, and imipenem-resistant nonfermentative bacteria. However, resistance is also a growing problem in community-acquired infections. Of particular concern are penicillin-resistant pneumococci (*3*–*5*) and the extended-spectrum beta-lactamase-producing enterobacteria (*6*). In addition, the World Health Organization (WHO) and the International Union against Tuberculosis and Lung Disease have shown that resistant tuberculosis (TB) is a problem in many parts of the world (*7*). Antimicrobial resistance often leads to therapeutic failure of empirical therapy; therefore, knowledge of the local prevalence of pathogens and their antimicrobial sensitivity patterns is essential for clinicians in their routine work. Clinicians should also be aware of the sensitivity patterns in both neighboring and distant areas.

In 1996, the Croatian Academy of Medical Sciences established the Committee for Antibiotic Resistance Surveillance in Croatia. The aims of the committee are to standardize methods for antimicrobial sensitivity testing in laboratories throughout the country, collect local data on antimicrobial resistance, and share the information with clinicians and pharmaceutical companies. The ultimate goal is a more appropriate use of antimicrobial agents in empirical therapy of infectious diseases. We present results of the surveillance for 1999 and describe the organization and activities of the committee.

## Methods

### Administration and Activities

**Figure 1:**
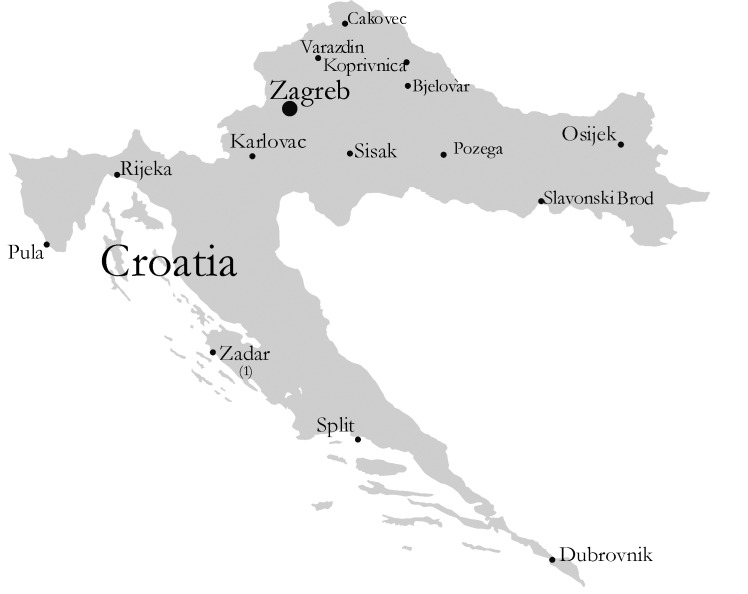
Croatian microbiology laboratories participating in surveillance of antimicrobial resistance

Seventeen microbiology laboratories, representing the major geographic regions of the country, were initially asked to join the committee. Membership is now open to all 32 laboratories in Croatia. By 1999, 22 laboratories had joined the committee (Figure 1). The committee also includes infectious disease and clinical pharmacology specialists who are interested in antimicrobial resistance.

The committee meets twice a year to identify pathogens and antimicrobial agents to be surveyed in the next study period (June 1 to December 31). A smaller working group is set up to organize surveillance and produce forms for data collection. The forms are sent to the collaborating centers, then returned to the working group for analysis. The committee also organizes a focused study of one particular clinical problem (e.g., a study on methicillin-resistant *Staphylococcus aureus* [MRSA] and two studies of blood-culture isolates). Each year the committee summarizes its results in reports sent to the collaborating centers and made available publicly.

### Educational Activities

The first Croatian Symposium on Antibiotic Resistance was organized in 1993. After the Committee for Antibiotic Resistance Surveillance was founded, it assumed responsibility for organizing these symposia every 3 years. The committee also organizes a biannual Workshop on Antibiotic Resistance at the Croatian Congress of Infectious Diseases. Each year, in collaboration with the local office of the Croatian Medical Association, the committee organizes a 1-day meeting on antibiotic resistance in different counties. In addition, the committee organizes an annual course on laboratory methods that is mandatory and free of charge for the heads of the collaborating laboratories.

### Quality Control

The Department of Microbiology at the University Hospital for Infectious Diseases in Zagreb participates in the WHO and Centers for Disease Control and Prevention External Quality Assurance program and acts as Croatian coordinator for member laboratories. This is the only organized quality assurance program in Croatia, and all committee members are required to participate. The laboratories are also encouraged to use the WHONET program for internal quality control.

## Surveillance and Laboratory Methods

The surveillance period was from June 1 to December 31, 1999. Organisms selected for surveillance included group A streptococcus, *Streptococcus pneumoniae, Staphylococcus aureus, Enterococcus* spp., *Escherichia coli, Klebsiella* spp., *Proteus* spp., *Enterobacter* spp., *Acinetobacter* spp., *Pseudomonas aeruginosa, Salmonella* spp., and *Shigella* spp. Laboratories were asked to record all nonduplicate isolates of these species during the surveillance period and their sensitivities to selected antimicrobial agents. The information was recorded on paper forms and returned to the working group. The isolates were collected from all body sites, and surveillance of routine data did not include differentiation of colonization from infection. Data for *Mycobacterium tuberculosis* were adapted from the annual report of the Croatian Reference Laboratory for Tuberculosis, which includes data from all laboratories in the country that process specimens for TB.

### Identification of Bacteria

All organisms were assessed by colony morphology and Gram stain. All the laboratories used the DNA-base and slide coagulase tests for identification of *S. aureus*, bacitracin disk for group A *Streptococcus*, and optochin disk for *S. pneumoniae*. Enterobacteria and *P. aeruginosa* were identified by different methods in different laboratories.

### Antimicrobial Sensitivity Testing

All participating laboratories used the disk diffusion method of the National Committee for Clinical Laboratory Standards for antimicrobial sensitivity testing (*8*). Disks for the selected antimicrobial agents were provided by the committee to ensure that all isolates were tested for all agents requested.

## Results

The centers providing data included 22 hospital and public health laboratories (Figure 1). Resistance rates for the organisms isolated in different centers varied widely, but certain trends were apparent.

### Gram-Positive Bacteria (Figure 2)

**Figure 2:**
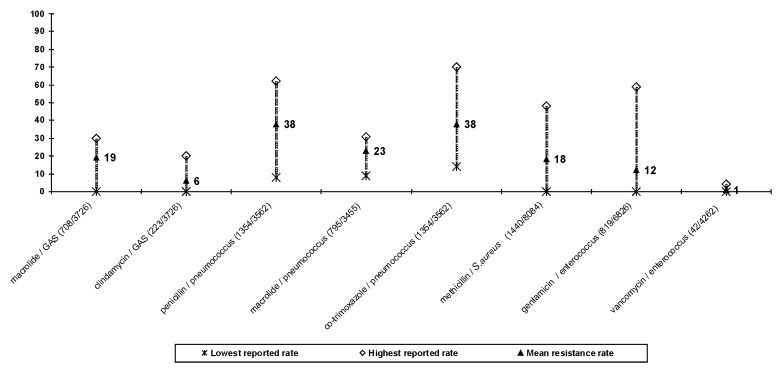
Resistance rates (%) to selected antimicrobial agents in gram-positive bacteria in Croatia (June 1 to December 31, 1999.) The number of resistant organisms / number of organisms tested is given in brackets. Note: macrolide resistance = resistance to erythromycin; azithromycin/penicillin resistance in pneumococci = nonusceptibility to penicillin; gentamicin resistance in enterococci = high-level resistance.

Among group A *Streptococcus* isolates, the overall resistance was 19% to erythromycin and 6% to clindamycin. *S. pneumoniae* isolates included those from primarily sterile sites, as well as from respiratory specimens and nasopharyngeal swabs. Resistance to penicillin and other antimicrobial agents was common. Both penicillin and cotrimoxazole resistance averaged 38%, macrolide resistance 23%, and tetracycline resistance 21%. Chloramphenicol resistance was relatively uncommon, averaging 7%.

These figures, however, obscure a wide range of results from different centers. Penicillin resistance ranged from 8% in Osijek to 62% in _akovec; erythromycin and azythromicin resistance from 9% in Dubrovnik to 31% in one Zagreb center; and cotrimoxazole resistance from 14% in Sisak to 70% in Pula.

Overall, 18% of Croatian isolates of *S. aureus* were resistant to oxacillin (and therefore methicillin). Methicillin-resistant strains were often multidrug resistant, averaging 65% resistance to gentamicin, 49% to clindamycin, 57% to ciprofloxacin, and 50% to rifampicin. Resistance to fucidic acid (5%), cotrimoxazole (7%), and mupirocin (7% when tested by a 5-mg disk) was uncommon. Again, wide variation in resistance rates was seen, with strikingly low rates of MRSA in the _akovec General Hospital.

Overall, only 2% of *Enterococcus* spp. isolates were resistant to ampicillin and 1% to nitrofurantoin. Resistance to gentamicin was 12% (when tested by a 120-mg disk), and vancomycin-resistant isolates occurred in only one Zagreb hospital (with 4% resistance).

### Gram-Negative Bacteria (Figure 3)

**Figure 3:**
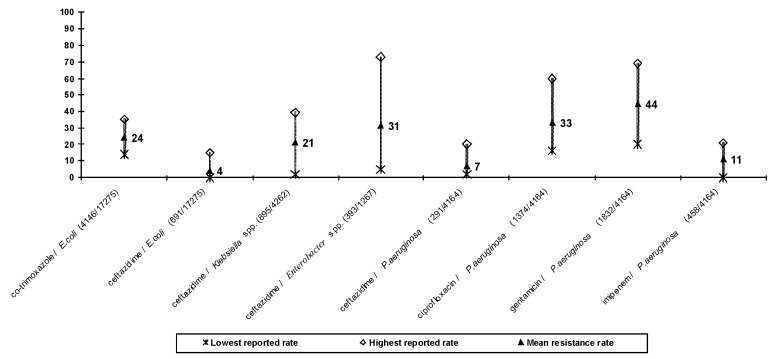
Resistance rates (%) to selected antimicrobial agents in gram-negative bacteria in Croatia (June 1 to December 31, 1999) The number of resistant organisms / number of organisms tested is given in brackets.

Approximately half the *E. coli* isolates were resistant to ampicillin, and 15% were resistant to beta-lactam/beta-lactamase inhibitor combinations. Approximately 24% of isolates were resistant to cotrimoxazole, 13% to cefuroxime, 4% to ceftazidime, 7% to gentamicin, 5% to ciprofloxacin, and none to imipenem. In almost all centers, resistance to ampicillin was >40% and to cotrimoxazole >20%. In one Zagreb hospital, resistance to ceftazidime reached 15%, but in most centers it was <4%.

*Klebsiella* organisms showed a high degree of multidrug resistance: 34% were resistant to co-amoxiclav or ampicillin+sulbactam, 33% to cefuroxime, and 21% to both ceftazidime and gentamicin. These organisms also showed moderate resistance (8% to 10%) to the other clinically available aminoglycosides (netilmicin and amikacin) but were generally sensitive to ciprofloxacin (6% resistance). No imipenem resistance was recorded. However, resistance rates varied widely, with ceftazidime resistance ranging from 2% to 39%, gentamicin resistance from 2% to 44%, and ciprofloxacin resistance from 1% to 17%.

Croatian isolates of *P. aeruginosa* also showed high rates of resistance and multidrug resistance, again with variation among centers. The overall rates of resistance (and ranges for different centers) were piperacillin 22% (7% to 57%), ceftazidime 7% (2% to 20%), imipenem 11% (0% to 21%), gentamicin 44% (20% to 69%), and ciprofloxacin 33% (16% to 60%).

Salmonellas generally showed good sensitivity to all antimicrobial agents except ampicillin (19% resistant) and chloramphenicol (6% resistant). Shigellas were highly resistant to ampicillin (87%), tetracycline (79%), and cotrimoxazole (89%). Resistance to other antimicrobial agents was <10%.

There was no imipenem resistance in enterobacteriaceae, but in *Acinetobacter* spp. it was 1% (0% to 8%). A high percentage (53% to 88%) of *Acinetobacter* isolates were resistant to many antimicrobial agents, except to the combination ampicillin + sulbactam (18% resistance), amikacin (25% resistance), and netilmicin (26% resistance). *Proteus* spp. isolates generally showed good sensitivity to antimicrobial agents except to ampicillin (49% resistance) and cotrimoxazole (28% resistance). *Enterobacter* spp. were often multidrug resistant, with stable resistance to ceftazidime of 31%.

In 1999, 5,664 isolates of *M. tuberculosis* were recovered in 17 laboratories; 316 (5.9%) of them were resistant to one of the first-line antituberculosis drugs (streptomycin, izoniazid, rifampin, pyrazinamid, or ethambutol). However, among newly diagnosed cases of TB, only 3% of isolates were resistant to one of the first-line drugs and 0.6% of isolates were multidrug resistant, i.e., resistant to rifampicin plus isoniazid.

## Discussion

Croatian national data suggest that resistance is occurring in both community- and hospital-acquired infections. *S. pneumoniae* is a major community-acquired pathogen. Resistance to penicillin of 38% has prompted an ongoing centralized study, which could also provide an estimate of the proportion of highly resistant strains.

MRSA is common, especially in large hospitals and on trauma wards. The incidence of MRSA increased rapidly during the early 1990s, frequently causing chronic osteomyelitis after war injuries. Observations in a trauma hospital in Zagreb suggest that MRSA spread throughout Croatia is facilitated by lack of screening and isolation facilities and poor interhospital communication (*9*,*10*). Some areas, such as the Medimurje region (city of _akovec), are still almost unaffected by MRSA, and screening of all surgery and intensive-care unit patients transferred there from other centers is highly recommended.

Croatia does not seem to have a nationwide problem with vancomycin-resistant enterococci. Such isolates appear to be limited to the Clinical Hospital Center Zagreb, the largest hospital in Zagreb (*11*). Cotrimoxazole is still widely used in Croatia as the first-line antimicrobial agent for urinary tract infections. In many centers, resistance of *E. coli* and other enterobacteriaceae to this agent exceeds 20%, which indicates the need for alternative therapy for uncomplicated urinary tract infections. Production of extended spectrum beta-lactamases (ESBL) in *E. coli* is still rare, except in the Clinical Hospital Center Rebro in Zagreb. The first outbreaks of ESBL-producing *Klebsiella* organisms were described in Europe in the mid-1980s (*12*), and by 1999 >30% of *Klebsiella* isolates from three of five Zagreb hospitals were resistant to third-generation cephalosporins. It is already common for Croatian isolates of *P. aeruginosa* to be resistant to aminoglycosides, but of new concern is the finding that resistance to imipenem reaches 20% in some centers. While shigellas are usually multidrug resistant, salmonellas generally show good sensitivity to antimicrobial agents, except to ampicillin. ESBL-producing salmonellas, described as causing outbreaks both in the community and hospitals (*6*,*13*,*14*), were identified in Croatia for the first time in 2000 (unpub. data).

Anti-TB drug resistance is a particular problem throughout the world, with multidrug-resistant TB in new cases reaching >2% in one third of all countries (*7*,*15*). With 0.6% of multidrug resistance in new cases, Croatia has a low incidence of multidrug-resistant TB. However, difficulties with isolating patients and fully implementing the directly observed treatment strategy (*16*) complicate the situation.

Apart from providing national data, setting up a surveillance program has heightened awareness of the problem of antimicrobial resistance throughout Croatia. Both local and national data are published in the committee’s annual report and distributed to the participating institutions. Such data are also discussed at a series of local meetings with clinicians and used as the basis for local antibiotic policies. The results of this nationwide surveillance serve as an early warning system for the emergence of antimicrobial resistance and indicate where more focused studies are needed.

The first year’s experience showed that educational and external quality control programs were needed to supplement the surveillance program. Training courses organized by the committee improved the standardization of the laboratory procedures throughout the country and had great impact on detection of resistance mechanisms and infection control. By taking part in the surveillance network, microbiologists were stimulated to discuss antimicrobial resistance and treatment with ward staff, pharmacists, and managers; sensitivity testing methods were reviewed; and technical staff were taught to flag the isolation of resistant bacteria. This process is ongoing.

Laboratory methods need to be kept under continual review, and introducing a national quality control scheme was very helpful. An ongoing surveillance program would be greatly facilitated by the use of microcomputers connected by e-mail for constructing and updating electronic databases. The committee also intends to initiate collaboration with the Croatian Ministry of Health to ensure that official policies favor appropriate use of antimicrobial therapy.

### Acknowledgments
